# Is location more determining than WHO grade for long-term clinical outcome in patients with meningioma in the first two decades of life?

**DOI:** 10.1007/s00508-024-02382-w

**Published:** 2024-05-31

**Authors:** Dorian Hirschmann, Danial Nasiri, Christian Joachim Entenmann, Christine Haberler, Thomas Roetzer, Christian Dorfer, Matthias Millesi

**Affiliations:** 1 Department of Neurosurgery, Medical University of Vienna, Vienna, Austria; 2 Department of Neurosurgery, Inselspital Bern, Bern, Switzerland; 3 Department of Neurosurgery, Charité Universitätsmedizin Berlin, Berlin, Germany; 4 Division of Neuropathology and Neurochemistry, Department of Neurology, Medical University of Vienna, Vienna, Austria

**Keywords:** Pediatric meningioma, Relapse, Adolescent, Skull base, Clear cell meningioma

## Abstract

**Objective:**

To identify factors for tumor relapse and poor outcome in patients with meningiomas in the first two decades of life.

**Methods:**

All patients ≤ 21 years of age who underwent resection of a meningioma at the department of neurosurgery, Medical University of Vienna between 1989 and 2022 were included in this retrospective study. Clinical and radiological data were extracted from the medical records. Outcome and tumor relapse were analyzed for tumor location, histological findings and extent of resection.

**Results:**

In this study 18 patients were included, 6 meningiomas were located in the skull base, 5 in the convexity and 7 in other locations including intraventricular and spine (2 patients each), falx, intraparenchymal and optic nerve sheath. Most frequent symptoms were seizures and cranial nerve palsy. In total 56% of the meningiomas were World Health organization (WHO) grade 1, 39% grade 2 and 5% grade 3. Gross total resection was achieved in 67%. The overall relapse rate was 61% and 50% underwent repeat surgery. All patients with convexity meningiomas became seizure free and had a favorable outcome. Relapse and clinical outcome were independent of WHO grade among the whole cohort but the outcome significantly depended on the WHO grade when patients with skull base meningiomas were analyzed as a subgroup. The relapse rate was significantly higher in cases of skull base location (100% vs. 42%, *p* = 0.038) and after subtotal resection (100% vs. 42%, *p* = 0.038). Clinical outcome was also significantly worse and the rate of complications was higher in patients with skull base meningiomas.

**Conclusion:**

Patients with convexity meningiomas in the first two decades of life have a good outcome due to high chance of gross total resection. Patients with skull base meningioma are at high risk of relapse and poor outcome, particularly those with WHO grades 2 and 3. Subtotal resection in patients with skull base location is probably the main reason for this difference.

## Introduction

Meningiomas in children and adolescents are a poorly understood tumor entity. Data are limited to small case series and meta-analysis. Most authors report the following key features of meningiomas in the first two decades of life: in comparison to their adult counterparts, meningiomas in children and adolescents are exceedingly rare and account for approximately only 1–3% of all intracranial tumors in this age group. Furthermore, male preponderance has been reported, which contrasts demographic data of adult meningioma patients [1, 2, 5, 10, 13, 15]. Studies about clinical long-term outcome and long-term radiological follow-up are rare but in general good results after resection of World Health Organisation (WHO) 1 and 2 tumors have been reported. A meta-analysis including 677 children indicated that the 15-year relapse rate of meningiomas even after gross total resection is as high as 21%, and up to 89% in cases of subtotal resection [8]. Children and adolescents with benign intracranial tumors are considered to have a long life-expectancy after resection. Thus, analysis of data about follow-up clinical status and tumor relapse is fundamental to assess patient long-term benefits from surgery. We therefore conducted this retrospective analysis of patients with meningiomas treated in the first two decades of life.

## Material and methods

### Study design

Patients ≤ 21 years of age who underwent surgery of a meningioma at the department of neurosurgery, Medical University of Vienna between 1989 and 2022 were included in this retrospective study. Histological diagnosis of a meningioma was confirmed in all patients. Demographic patient data, radiological and histological findings, and clinical preoperative and postoperative data were retrospectively extracted from clinical records. Radiology reports were reviewed in cases of missing imaging data.

### Treatment

Based on the low age of this patient group and a good prognosis of a presumably benign tumor entity, the general aim of surgery was total resection. As documented in the operative reports, the extent of resection (EOR) was graded according to the Simpson classification, with gross total resection (GTR) being defined as grade I or II [6]. After confirmation of the histological diagnosis each case was discussed in a multidisciplinary board to evaluate the necessity of further treatment and follow-up examinations.

### Clinical outcome evaluation and follow-up

Functional outcome was assessed by the Karnofsky performance status scale (KPS) and seizure outcome according to the Wieser classification in cases of preoperatively diagnosed seizures [23]. The clinical status at time of last follow-up was compared to the preoperative status in every patient. Complications were defined as events associated with surgery, which led to reoperation, increase of length of stay or permanent new neurological symptoms. Favorable clinical outcome was defined as KPS ≥ 80. In cases of secondary clinical deterioration due to untreatable tumor relapse, the time from surgery to significant neurological decline was documented.

### Radiological follow-up

Radiological follow-up included regular performance of magnetic resonance imaging (MRI) examinations once a year after surgery. According to the radiological findings, the presence or absence of a remnant or tumor recurrence was documented. Recurrence after GTR and remnant growth are referred to as relapse.

### Histopathological analysis

Histological material of all cases included in the series was revised by two pathologists and findings were updated according to WHO 2021 classification. In addition, cases with clear-cell appearance were immunohistochemically stained with anti-SMARCE1 (HPA003916, Lot#:A107052, Sigma-Aldrich, St. Louis, MO, United States) to confirm loss of nuclear SMARCE1 expression.

### Data analysis

Data are presented as counts and percentages or as median and range. Categorical variables were analyzed by χ^2^-tests. Mann-Whitney U‑test was used to identify differences between metric variables. Differences between preoperative and postoperative modified Rankin Scale (mRS) scores were assessed using the Wilcoxon signed-rank test. Actual rates of relapse-free survival (RFS) were calculated by Kaplan-Meier survival analysis and compared with the Breslow test. Statistical analyses were conducted using IBM SPSS Statistics for Windows (version 24 IBM, Armonk, NY, USA) with the significance level set to α = 0.05. A central death register comparison was performed via Statistic Austria (Bundesanstalt Statistik Österreich, Guglgasse 13, 1110 Wien).

### Ethical statement

This study was approved by the Institutional Ethics Committee (EK 1856/2022) and complies with the principles of the Declaration of Helsinki.

## Results

### Study population

Between 1989 and 2021, a total of 18 patients ≤ 21 years of age underwent resection of a meningioma at our department. Table 1 shows the baseline characteristics of the cohort with a median age of 15.5 years and a male:female ratio of 10:8. One patient with a large skull base meningioma was 21 years and 2 months of age at the time of surgery; however, onset of symptoms was 7 months before surgery, so the patient was included. The most frequent locations of meningiomas were the skull base (33%) and convexity (27%), followed by intraventricular and spinal tumors, whereas meningiomas rarely originated from other locations, such as the falx, parenchyma or optic nerve sheath. Three patients had a history of prior radiation exposure at the site of the tumor, one located at the falx, one at the convexity and one spinal at the C4 level. Multiple meningiomas were present in three patients, one of which was diagnosed with neurofibromatosis II (NF II).

**Table 1:** Baseline characteristics of 18 patients who underwent resection of a meningioma

	*n* = 18
*Median age in years (range)*	15.5 (2–21)
*Male sex*	10 (56%)
*Tumor location*	
Skull base (bone)	6 (33%)
Convexity	5 (27%)
Intraventricular	2 (11%)
Spinal	2 (11%)
Falx	1 (6%)
Intraparenchymal	1 (6%)
Optic nerve sheath	1 (6%)
Median tumor size in mm (range)	30 (10–90)
*Main symptoms at diagnosis*	
Seizures	6 (33%)
Cranial nerve palsy	4 (22%)
Visual impairment	3 (17%)
Headache	3 (17%)
Signs of elevated ICP	3 (17%)
None	2 (11%)
*Median KPS score at time of diagnosis (min–max)*	80 (20–100)
*Multiple meningiomas*	3 (17%)
*NF II*	1 (6%)
*Prior radiation exposure*	3 (17%)
*Number of resections*	
One resection	8 (45%)
Two resections	9 (50%)
Three resections	1 (5%)

### Clinical presentation

The most common symptom at time of diagnosis were epileptic seizures (33%), which was clearly associated with convexity location (*p* = 0.025) and 4 patients (22%) presented with cranial nerve palsy. Other clinical findings were headache, signs of increased intracranial pressure (ICP) and absence of neurological symptoms as listed in Table 1. The median KPS at presentation was 80 (20–100).

### Treatment

Data on treatment and outcome are shown in Table 2. All 18 patients underwent resection of a meningioma, in most of the patients (67%) GTR (i.e., Simpson grade I or II) resection was documented in the operating report and Simpson grade IV in the remaining 6 cases (33%); however, 9/18 patients (50%) underwent a second resection due to tumor relapse and 1 patient (5%) had a third resection. Two patients with relapse did not undergo a second operation, one patient rejected further surgery and one was treated with gamma knife radiation only. In half of the cases of skull base meningiomas, Simpson grade IV only was achieved compared to grade I in all cases of convexity meningiomas. In cases of subtotal resection, adherence to major blood vessels and/or brain stem was documented in the operative report.

**Table 2:** Summary of the most important preoperative and postoperative data including clinical outcome

Location	Location detailed	WHO grade	Histology	Simpson grade	History of radiation	Recurrence/remnant growth	Number of resections	KPS preoperative and last FU	Seizures preoperative and last FU
6 Skull base	Petroclival	WHO 1 → WHO 3	Meningotheliomatous → Anaplastic	IV	No	Yes	2	90 → 0	No → Yes^a^
	Diaphragma sellae	WHO 1	Meningotheliomatous	II	No	Yes	2	*90* *→* *90*	No → No
	Frontobasal	WHO 1	Meningotheliomatous	II	No	Yes	2	*90* *→* *80*	No → No
	Petroclival	WHO 2	Clear cell	II	No	Yes	2	80 → 0	No → No
	Clivus	WHO 1	Transitional	IV	No	Yes	2	80 → 30	No → No
	Petroclival	WHO 2	Clear cell	IV	No	Yes	3	70 → 0	No → No
5 Convexity	Parietal	WHO 1	Fibroblastic	I	No	**No**	1	***90*** ***→*** ***100***	**Yes** **→** **No**
	Parieto-occipital	WHO 1	Fibroblastic	I	No	**No**	1	***50*** ***→*** ***80***	**Yes** **→** **No**
	Parietal	WHO 2	Atypical	I	No	Yes	2	***60*** ***→*** ***100***	No → No
	Temporal	WHO 2	Atypical	I	No	**No**	1	***90*** ***→*** ***100***	**Yes** **→** **No**
	Frontal	WHO 2	Atypical	I	Yes	**No**	1	***50*** ***→*** ***100***	**Yes** **→** **No**
2 Intraventricular	3rd Ventricle	WHO 1	Fibroblastic	IV	No	Yes	1	***80*** ***→*** ***100***	No → No
	Lateral Ventricle	WHO 2	Atypical	I	No	Yes	2	20 → 0	No → Yes
2 Spinal	C2	WHO 1	Psammomatous	I	Yes	**No**	1	*100* *→* *100*	No → No
	Th5	WHO 1	Meningotheliomatous	I	No	**No**	1	60 → 70	No → No
1 Falx	Frontal	WHO 2	Atypical	I	Yes	**No**	1	*100* *→* *100*	No → No
1 Intraparenchymal	Frontal	WHO 1	Fibroblastic	IV	No	Yes	1	***90*** ***→*** ***100***	Yes → Yes
1 Optic nerve sheath	Cisternal segment	WHO 1	Meningotheliomatous	IV	No	Yes	2	*80* *→* *80*	No → No

*Italics* data indicate favorable outcome and **bold** data indicate improvement compared to preoperative status.

^a^Patient had seizures at time of last alive follow-up (*FU*)

In total 8 patients (44%) experienced surgery-related complications, including 4 cases of shunt-dependent hydrocephalus, 1 wound infection, 1 postoperative hemorrhage and 5 cases of new neurological deficits after surgery. Of note, all 4 cases of postoperative shunt-dependent hydrocephalus occurred in patients with skull base meningiomas and in 1 patient with an intraventricular meningioma. Furthermore, 4 of 5 cases of new neurological symptoms were assigned to skull base meningiomas and 1 case to an optic nerve sheath meningioma. One case of hemorrhage was documented in a spinal meningioma. One patient with a skull base meningioma had to undergo revision surgery due to wound infection. There were no complications in patients with convexity meningiomas. The difference in incidence of complications between skull base and other location was significant (odds ratio [OR] 15.0, 95% confidence interval [CI] 1.2–185.2, *p* = 0.043, see Table 3).

**Table 3:** Differences in the incidence of complications and in median preoperative and postoperative KPS between skull base and other locations as well as suspected factors associated with tumor recurrence

*n* = 18			*p*
–	Skull base	Other locations	–
*Complications*			
Complication	5/6 (83%)	3/12 (25%)	**0.043**
No complications	1/6 (17%)	9/12 (75%)	
*Median KPS (min–max)*			
Preoperative	85 (70–90)	80 (20–100)	0.553
At last FU	15 (0–90)	100 (0–100)	**0.013**
*Median KPS according to WHO grade (min–max)*			
WHO I	80 (30–90)	–	**0.037**
WHO II-III	0 (0–0)	–	
WHO I	–	100 (70–100)	0.628
WHO II-III	–	100 (0–100)	
–	Relapse	No relapse	–
*Location*			
Skull base	6/6 (100%)	0/6	**0.038**
Other location	5/12 (42%)	7/12 (58%)	
Convexity	1/5 (20%)	4/5 (80%)	**0.047**
Other location	10/13 (77%)	3/13 (23%)	
*Extent of resection*			
Simpson I	5/12 (42%)	7/12 (58%)	**0.038**
Simpson IV	6/6 (100%)	0/6	
*WHO grade*			
WHO 1	6/10 (60%)	4/10 (40%)	0.709
WHO 2	4/7 (57%)	3/7 (43%)	
WHO 3	1/1 (100%)	0/1	

**Bold** type figures indicate significant *p*-values.

Of the patients 2 (11%) received radiotherapy, 3 (17%) received gamma knife treatment, and 2 patients (11%) underwent chemotherapy in addition to surgery including bevacizumab, gemcitabine, interferon and trabectedine. One patient (5%) underwent implantation of iodine-125 seeds. The indications for such adjuvant treatments were limited to inoperable remnant or relapse of an atypical or anaplastic meningioma.

### Long-term clinical outcome

The median overall time of follow-up (FU) was 123 months (range 26–335 months) and no patients were lost to FU. The overall median KPS at time of the last FU was 85 (0–100). In total 4 patients died according to a death register comparison. All of the 4 patients died due to relapse of a tumor WHO grade 2 or 3. Of note, clinical outcome was clearly dependent on tumor location: As depicted in Table 2 none of the patients with a skull base meningioma improved in KPS score during the observation period, compared to all of the patients with a convexity meningioma. Conversely, the overall KPS of patients with skull base meningioma substantially declined during follow-up. The median KPS score at time of last FU in skull base meningioma patients was 15 (0–90) compared to a median score of 100 (0–100) in patients with tumors in other locations (*p* = 0.009). This difference was not seen preoperatively, when patients in the skull base group had a median KPS score of 85 (70–90) compared to 80 (20–100) in the other patients (*p* = 0.553), see Table 3. In total, 6 of 18 patients (33%) eventually had an unfavorable outcome (= KPS < 80); however, 3 of these patients initially had a favorable outcome but then deteriorated slowly and gradually during follow-up, starting after a median time of 145 months (80–309 months) after surgery due to an inoperable tumor relapse. One patient with unfavorable outcome had NF II and suffered preoperatively from multiple cranial nerve palsies due to large bilateral vestibular schwannomas. Hence, the patient was not able to improve in KPS after resection of the meningioma. Furthermore, unfavorable outcome in 2 patients (KPS 30 and 70) resulted from complications leading to new neurological symptoms after surgery.

### Seizures

In total, 6 patients (33%) suffered from seizures before surgery, 4 of whom were patients with convexity meningioma. All patients with convexity meningioma were seizure-free (Wieser class I) at time of last FU. In total, 3 patients suffered from seizures at time of last FU, including 2 cases (WHO 2 and 3) who developed de novo seizures during follow-up and one case of a recurrent intraparenchymal meningioma which was classified Wieser class IV.

### Histological findings

The WHO grades and specific histological diagnoses are listed in Table 2. In total, 56% were classified as WHO 1, 39% WHO 2 and 5% WHO 3 as per last histological diagnosis. Immunostaining revealed SMARCE1 mutations in both cases of clear cell meningiomas. When the two subgroups skull base and convexity meningioma were compared, the histological subtype was associated with tumor location (*p* = 0.012). Thus, tumors located in the skull base were primarily either classified as meningotheliomatous (WHO 1) or clear cell meningiomas (WHO 2). In contrast, histological findings of convexity meningiomas revealed either a fibroblastic (WHO 1) or atypical (WHO 2) subtype. Furthermore, as shown in Table 2, clinical outcome in all patients with convexity meningiomas was favorable, regardless of the WHO grade (*p* = 0.628). In contrast, clinical outcome was unfavorable in patients with skull base meningioma of WHO grade 2 or 3 but favorable in those with WHO grade 1 (*p* = 0.037).

Of note, in one case, which was diagnosed with a meningotheliomatous subtype (WHO 1) after first and second surgeries, transformation into an anaplastic meningioma (WHO 3) had been observed 23 years after initial surgery, when a biopsy was done.

### Tumor relapse after first resection

**Fig. 1:**
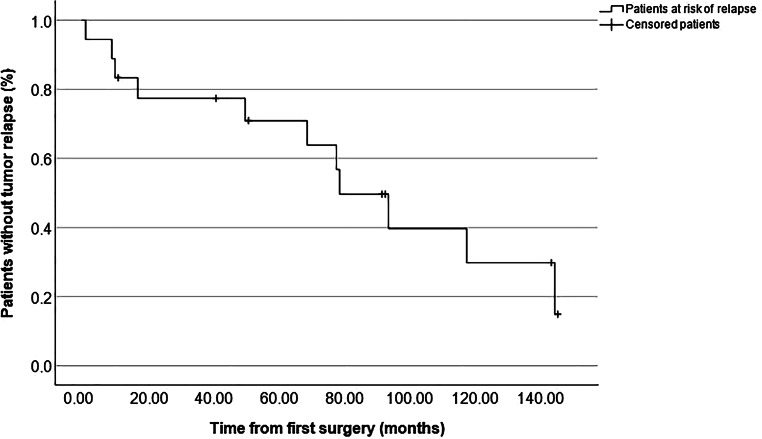
Graph showing relapse-free survival of the whole cohort with an actuarial median time to relapse of 79 months (55–103 months)

**Fig. 2:**
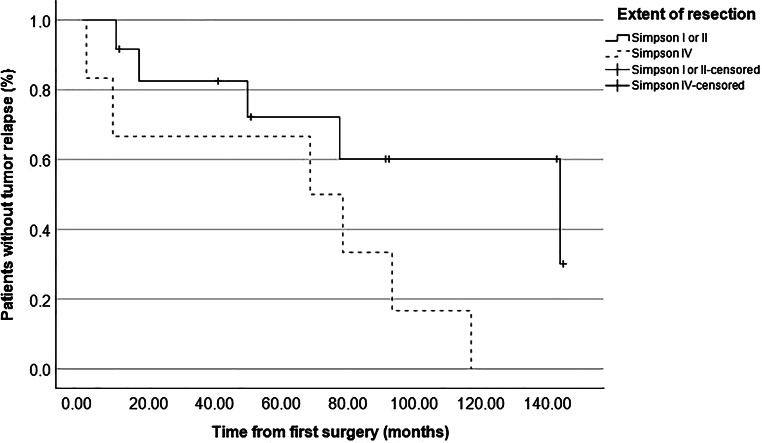
Graph showing relapse-free survival according to extent of resection. A trend could be observed towards earlier relapse in patients who underwent subtotal resection (median time to relapse 69 months vs. 145 months, log-rank: 0.062)

**Fig. 3:**
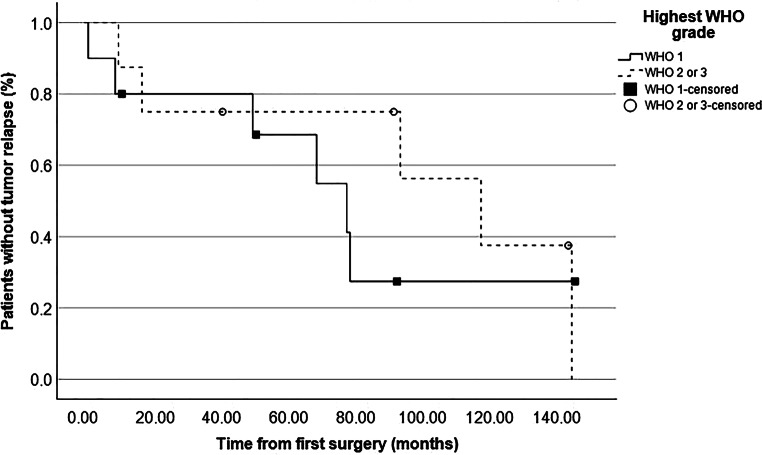
Graph showing relapse-free survival according to WHO grade. No difference was observed between the two groups (78 vs. 118 months, log-rank: 0.507)

**Fig. 4:**
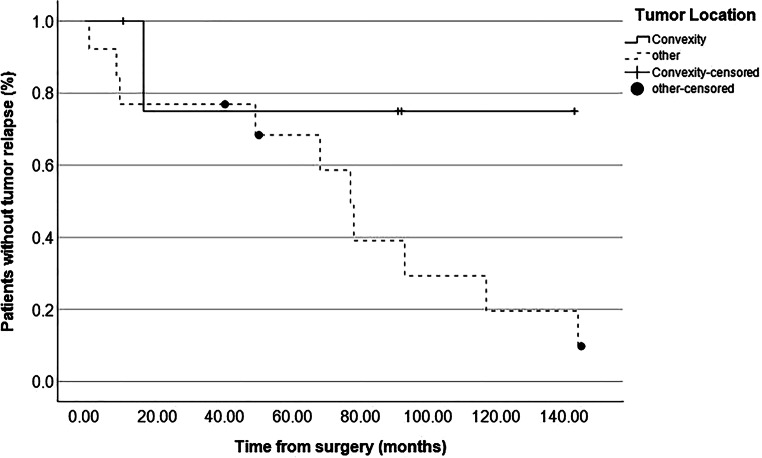
Graph showing relapse-free survival in patients with convexity meningiomas compared to other locations. Median time to relapse was 78 months (63–93 months) in patients with locations other than convexity. No median time to relapse is given for convexity meningiomas. According to log-rank test, no significant difference was seen (*p* = 0.187)

The median time from surgery to last radiological FU was 109 months (11–335 months) and no patient was lost to radiological FU. In total, in 11 of 18 patients (61%) tumor relapse was seen during follow-up after the initial resection. Hence, in these cases, either growth of a remnant or recurrence after gross total resection (GTR) were documented. As shown in Table 3, relapse occurred significantly more often in cases of skull base location (*p* = 0.038) compared to other locations and significantly less often in cases of convexity location (*p* = 0.047) compared to other locations. Furthermore, relapse among the whole cohort was significantly associated with subtotal resection, i.e., Simpson grade IV (*p* = 0.038) but not with higher WHO grades (*p* = 0.709). Figure 1 shows the overall relapse-free survival with a median time to tumor recurrence of 79.0 months (39.9–118.1 months). Actual rates of relapse-free survival according to Simpson grade, WHO grade and tumor location are depicted in Figs. 2, 3 and 4, respectively, showing a trend but no significant difference in the former (*p* = 0.062) and no association in the latter two (*p* = 0.507 and *p* = 0.187).

### Repeat surgery and long-term relapse

As shown in Table 2, half of the patients had to undergo repeat surgery due to relapse. After last surgery, at time of last follow-up, relapse was seen in three of those patients (33%). In all of these cases gross total resection was not possible due to infiltrative tumor growth affecting structures such as cavernous sinus, brain stem and the middle cerebral artery. In two of the patients, further surgery was abandoned due to poor clinical status; however, one patient received adjuvant gamma knife treatment after second surgery. The third patient who had an intraparenchymal meningioma, refused to undergo repeat surgery for personal reasons.

## Discussion

### Study population

The distribution of sex and age in the current study is in line with other reported series [8]. Our cohort includes 3 patients with a history of irradiation with 2 meningiomas of the convexity and 1 spinal meningioma. Indications for irradiation were lymphoproliferative disease in one case and atypical teratoid-rhabdoid tumor in two cases (CPA and 4th ventricle). Prior irradiation is a well-documented risk factor for meningiomas [19]. Another reported risk factor for meningiomas is NF II [20]. In our cohort one patient with bilateral vestibular schwannoma and multiple meningiomas was diagnosed with NF II, which was confirmed by genetic analysis. Another patient with a unilateral vestibular schwannoma and multiple meningiomas was highly suspicious of NF II but genetic analysis was not conducted. Hence, the rate of NF II in this cohort is 5–10%, which corresponds to the rates reported in the literature.

### Long-term clinical outcome

In our cohort patients with skull base meningioma had significantly worse clinical outcome than those with meningioma in other locations, especially when the outcome was compared to those of patients with convexity meningiomas. This is explainable by reduced accessibility and closer relation to cranial nerves and brain stem; however, comparable data on the postoperative outcome in this subgroup are scarce and existing case series do not compare outcomes between different locations [1, 5, 8, 10, 13]. Furthermore, in some cases of skull base meningiomas, radical gross total resection does not appear reasonable due to extensive involvement of vital structures, such as major blood vessels, cranial nerves or the brain stem. In these cases surgery aims at tumor reduction, decompression and histological diagnosis.

Of the skull base meningioma patients in our cohort, 3 who had died and 1 with a KPS score of 30 at time of last follow-up, had presented with good or acceptable neurological function for several years during follow-up. On the contrary, neurological deterioration in another patient with a skull base meningioma was clearly due to intraoperative complications. In a meta-analysis including 518 pediatric and adolescent meningioma patients published by Kotecha et al. 6.9% died due to tumor relapse and another 3% died due to complications of the resection. The reported outcome varies substantially between series (mortality 0–59%) and may depend on different baseline characteristics of treated patients [1, 2, 5, 8, 15]. Consequently, our relatively large share of patients with unfavorable outcome may be explained by their tumor characteristics.

### Histological findings

In comparison to other reported cohorts, the share of WHO 2 and 3 tumors in the current cohort was substantial, accounting for 50% of cases. In total, no difference in clinical outcome could be observed between patients with tumors of WHO grade 1 and 2 which is in line with existing meta-data [8]; however, when the cohort was classified according to tumor location, a significant difference in clinical outcome between WHO grades could be seen in patients with skull base meningiomas, but not in those with tumors located in the convexity. Hence, patients with skull base meningiomas of WHO grade 2 and 3 had the worst clinical outcome. Given the very limited sample size, this finding is remarkable. Comparison with the existing literature is difficult as clinical outcome dependent on WHO grade is usually not given for separate tumor locations. Furthermore, histological subtype may also be crucial for long-term outcome. Of six cases of skull base meningiomas in our series two were clear cell meningiomas (CCM) and one case transformed from WHO grade 1 to grade 3. All of these three patients died. These cases represent more aggressive subtypes with high reported recurrence rates up to 61%. Specifically, intracranial CCM are reported to behave more aggressively than other WHO 2 meningiomas [2, 14, 17, 22].

Another finding in our study was that the distribution of histological subtypes differed among the two locations skull base and convexity. Hence, skull base meningiomas were primarily of either a meningotheliomatous or clear cell subtype, whereas the convexity counterparts were diagnosed as fibroblastic or atypical subtypes. This finding may suggest different specific cellular or pathway origins of meningiomas dependent on their anatomical location but data addressing this question within the age group of children and young adults are scarce; however, in adults in accordance, correlations between genetic alterations and meningioma location have been reported, also suggesting an association between cell origin and tumor location. Kros et al. found that convexity meningiomas are more likely to have NF2 mutations than meningiomas of the anterior skull base and Clark et al. reported correlation of mutated NF and/or chromosome 22 loss with tumor localization in the hemispheres [3, 9]. Furthermore, other authors have shown that using techniques of molecular pathology, including methylation profiling, whole exome sequencing and mRNA sequencing, enables clustering with more adequate classification concerning location as well as clinical prognosis than the WHO grading [4, 12, 21]; however, in the pediatric population meningiomas appear to be characterized by molecular features distinct from adult tumors. Hence, Kirches et al. have applied molecular pathological techniques to their pediatric cohort and found a different clustering within this age group in comparison to an adult dataset. Their cohort comprised of 37 meningiomas of which 36 were located intracranially [7]. In contrast, our cohort is smaller and less homogeneous. As molecular pathological techniques with subsequent (unsupervised) clustering require larger or at least more homogeneous cohorts, this approach does not appear to be promising in the case of our series.

Of note, one of the two cases of WHO 3 meningioma was diagnosed as WHO 1 at first resection, thus, this case resembles a rare case of malignant transformation. Malignant transformation of meningiomas has been reported with an incidence of 2.98/1000 patient years in a systematic review by Nakasu et al. with a median time of 5 years to transformation [11].

### Tumor relapse

The total rate of tumor relapse (i.e., recurrence or remnant growth) after first resection in the current study was 61%. We found two factors clearly associated with tumor relapse: subtotal resection and skull base location. In contrast there was no association between WHO grade and tumor recurrence among the whole cohort. Kotecha et al. reported in their meta-analysis a total tumor recurrence rate of 21% with respective rates ranging from 0% to 50% between different studies. In line with our findings, the authors reported a significant association between extent of resection and relapse as well as no difference in relapse-free survival (RFS) between WHO 1 and 2 meningiomas. On the contrary, RFS was reported to be significantly shorter in patients with WHO 3 meningiomas; however, our cohort included only one case of a WHO 3 tumor, hence, a reliable statistical analysis could not be conducted for this group. In contrast to our finding that relapse rate is independent of WHO grade, two studies found a significant difference in tumor relapse between WHO grades 1 and 2. Although the two cohorts include considerable numbers of patients (115 and 87 cases), their main weakness is their limited time of follow-up. Median time of follow-up was 27.6 months in one study and 68.6 months in the other [18, 20]. According to the metadata of Kotecha et al., median time to relapse was 43.2 months, hence, a longer time of follow-up may be necessary to sufficiently detect relapse. The median time of follow-up was 123 months in our study, which may allow a more representative assessment of tumor relapse. The importance of the duration of follow-up is confirmed by the series of Rochat et al. which included 22 children with a mean follow-up of 16 years (1–45 years) and revealed a 47% relapse rate and 59% mortality, although 91% were reported to be low grade meningiomas [16].

### Limitations

Our study and findings are limited by the small case number of our series which is due to the low prevalence and incidence of meningiomas of the first two decades of life. Furthermore, the retrospective and monocentric design of the study are obvious limitations. It has to be mentioned that published series include very inhomogeneous patient population concerning histological findings, in particular histological subtypes as well as tumor location. Hence, the possibility to compare different cohorts within the existing literature is limited. No molecular pathological techniques were applied in our study, hence, diagnoses are based on histological examinations according to the WHO 2021 classification and SMARCE1 immunostaining. The main reason for this was the fact that our series is small and inhomogeneous and thus clustering according to molecular features did not seem achievable; however, future investigations including multicenter molecular analyses of a larger cohort will be aimed for, to identify potential drivers of tumor growth and to allow more reliable differentiation of subtypes.

## Conclusion

Clinical outcome and risk of tumor relapse after resection of meningiomas in the first two decades of life are dependent on tumor location. Whereas convexity meningiomas of both WHO grades 1 and 2 appear to have a benign course after resection with a low relapse rate and good clinical outcome, meningiomas located in the skull base have a high risk of unfavorable outcome and relapse. This seems to apply particularly to lesions higher than WHO grade 1. Whether the reason for this is lower rates of gross total resection in skull base meningiomas compared to convexity location or a more aggressive nature of tumors which arise from the skull base is unclear. Gross total resection is still an important prognostic factor.
